# Oral-health-related quality of life in adolescents: umbrella review

**DOI:** 10.1186/s12889-023-16241-2

**Published:** 2023-08-23

**Authors:** Ítalo Gustavo Martins Chimbinha, Brenda Nayara Carlos Ferreira, Giovana Pessoa Miranda, Renata Saraiva Guedes

**Affiliations:** Dentistry Department, Federal University of Rio Grande Do Norte, Natal, Brazil

**Keywords:** Self-perception, Quality of life, Oral conditions, Children, Adolescents

## Abstract

**Background:**

To evaluate oral conditions, demographic and socioeconomic characteristics of oral health-related quality of life (OHRQoL) in adolescents.

**Methods:**

Umbrella review, conducted according to the Preferred Reporting Items for Systematic Reviews and Meta Analyzes (PRISMA) checklist. The search strategy used a combination of words, applied in the electronic databases PubMed, WebScience, Embase, Lilacs, Scopus and Cochrane. Included publications until January 2022, without restrictions. Data collection took place with systematized practices and the eligibility criteria were studies focusing on OHRQoL; teenagers; adolescentes; present the term “systematic review” and/or “meta-analysis” in the title or abstract. The quality assessment followed the Assessment of Multiple Systematic Reviews (AMSTAR 2) and the adherence of the article to the PRISMA was verified.

**Results:**

Three hundred sixty-two articles were identified, and 22 were included, published between 2009 and 2022. 21 Systematic reviews focused on the English language. Most studies showed heterogeneity in the methodological structuring process: 10 articles were considered of low and 10 critically low quality. Clinical conditions associated with worsening in quality of life were dental caries, malocclusion, dental trauma, toothache, edentulism, need for orthodontic treatment, irregular brushing, and periodontal disease. Socioeconomic factors related to housing, parental education, access to health care, absence of siblings and nuclear family influence OHRQoL. Completion of orthodontic treatment, health promotion programs, dental care and safe housing all have a positive impact.

**Conclusion:**

Worse oral health status, older age, female sex and worse socioeconomic status were significantly associated with worse OHRQoL.

**Trial registration:**

PROSPERO CRD4202129352.

## Background

In the last years, the interest in associating Quality of Life and Oral Health increased potentially. Quality of life is an individual's perception of their position in life in the context of the culture and value systems in which they live and in relation to their goals, expectations, standards and concerns. Oral health is the state of the mouth, teeth and orofacial structures that enables individuals to perform essential functions such as eating, breathing and speaking, and encompasses psychosocial dimensions such as self-confidence, well-being and the ability to socialize and work without pain, discomfort and embarrassment. Oral conditions and self-perception can impact the daily life and well-being of the individual [1–3] and are not restricted to physical effects, but associate family, social, economic, psychological, spiritual and environmental issues, depending on the accumulated risk throughout life [4].

In this context, adolescence is a period of vulnerability and involves hormonal, behavioral and psychological changes. Studies indicate changes in eating habits and aesthetic perception [5, 6]. Adolescents have specific needs and concerns that can cause oral disease [6]. In this age group Malocclusion gingivitis and periodontal disease are very common problems. Such as dental caries and DMFT [6–8].

The literature report worse oral health conditions impact school performance and socialization [5]. Impact on oral health-related quality of life (OHRQoL) increases proportionally with the severity of oral diseases [9, 10]. Fluorosis and dental caries impact on self-perception [11–13]. Pain and aesthetic problems are associated with the worst OHRQoL reports and greatest impact on social and emotional domains [13, 14]. Socioeconomic and behavioral factors are reported to be strong predictors for the impact on OHRQoL. Maternal education level, family income and social support can significantly influence the adolescent's self-perception [15].

However, other studies observed that adolescents do not benefit from health care and attention, when compared to children and adults [15, 16]. Health practices, stress mechanisms, need for treatment, resistance to dental consultations, fear and anxiety about dental care are possible factors that impact health-related quality of life in teenagers, but they are rarely reported in the literature [11, 17–19].

Systematic reviews carried out to observe the methodological quality of the studies, and thath encourage the realization of new, well-designed research on the subject [9, 10]. This because, many studies report general limitations in the papers included, which may compromise the quality of the evidence of the findings. Disagreements between authors on the method of evaluating predictive factors such as caries, frequency of dental visits and the outcome related to OHRQoL [9, 20], and the allocation of children and adolescents in the same group/assessment method may suggest biased data [9]. They also indicate the importance of using validated and tested socio-dental measures in different populations to analyze the impact on OHRQoL in adolescents [9, 10]. Untill now, only two instruments assess the impact of oral health on the oral health-related quality of life of adolescents between 11 and 18 years of age: the Caregiver Perceptions Questionnaire (CPQ 11–14), and the Child Oral Impacts on Daily Performances (Child OIDP [2, 19].

Therefore, the objective of this study is to review the literature who investigates the possible relationship of oral health conditions, demographic, socioeconomic and behavioral characteristics with OHRQoL in adolescents, through an umbrella systematic review.

## Methods

### Protocol and registration

For this umbrella systematic review the preferred reporting steps for systematic reviews and meta-analyses (PRISMA) [21] were followed, conducted in accordance with this checklist. A public search protocol was submitted to the International Prospective Registry of Systematic Reviews (PROSPERO) under registration number: CRD42021293528.

### Selection criteria

For this research, the inclusion criteria was articles characterized as a systematic review, with or without meta-analysis, without restriction of year of publication and language, which address the correlation between oral health conditions and a possible impact on quality of life in adolescents, of both sexes, 10 to 19 years old. Age established according to World Health Organization standards.

Systematic reviews, which included within their sample composition, individuals in a condition of vulnerable health, as well as pregnant women, or those in a situation of confinement/incarceration and indigenous people was excluded.

### Information sources, search protocol and search strategy

The search strategy involved the identification of keywords, which were used in the electronic databases, in order to identify all studies that address the relationship: oral condition and impact on quality of life of adolescents.

- The search strategy used for the Medline (PubMed) was:

- 1. ((Adolescents [MeSH]) OR (teenagers) OR (adolescence)) AND ((oral health [MeSH]) OR (mouth diseases [MeSH]) OR (oral health determinants)) AND ((quality of life [MeSH]) OR (OHQoL) AND ((systematic review).

This strategy was adapted to different databases, in accordance with their algorithms. The search included sevem databases: Medline (via PubMed), Embase, Scopus, Web of Sciences, Lilacs, Scielo and Cochrane, as well as the consensus between the evaluators and the consultation with the expert. In a period of twho weeks.

### Selection of studies and calibration of evaluators

The study selection process followed the PRISMA guidelines [21]. The results obtained from the search performed in the five consulted databases (Medline, via PubMed), Embase, Scopus, Web of Sciences, Lilacs and Cochrane) were exported to the Endnote™ X8.2 [22]. A database was created to facilitate the management and verification of duplicate articles.

Three independent reviewers (IGMC; BNCF; GPM) were previously trained and calibrated about the inclusion/exclusion criteria for the analysis of studies, through a pilot inclusion/exclusion round, analyzing the title and abstract of the articles obtained in the Search from PubMed. The article were read in full, if they did not provide enough information in the abstract. In this process, there was 100% consensus among the evaluators (Kappa = 1,0 high agrement).

The phase I of data extraction selected all sistematic reviews obtained through an Excel™ file. The document was filled with data: article title, author, year of publication, journal, specialty, systematic review study, presence of meta-analysis and population studied (age of adolescents).

### Selection of studies and data extraction

In the pahse I the reviewers (IGMC; BNCF; GPM) independently identified potential references, based on the title and abstract. In phase II, the articles were reed in full and irrelevante studies were exclued based in previously established criteria. The reason for exclusion of each article was documented.

In the next round, the selected articles were submitted to AMSTAR 2, a checklist composed of 16 items, with the objective of evaluating systematic reviews. Three reviewers (IGMC; BNCF; GPM) independently extracted relevant information: author, article name, year of publication, journal, outcome, independent variables, questions regarding the introduction, eligibility criteria, characteristics of the selected studies, quality analysis, risk of bias, presence of limitations and meta-analysis [23–25].

Also, the included studies had their references list manually checked by all reviewers to ensure the inclusion of possible works relevant to this topic.

Any source of conflict, throughout this process, was discussed until a consensus was reached. In case of discrepancy, a fourth reviewer was called. In addition, the authors were contacted in situations where the full article could not be obtained, or for clarification of information.

## Result

**Fig. 1:**
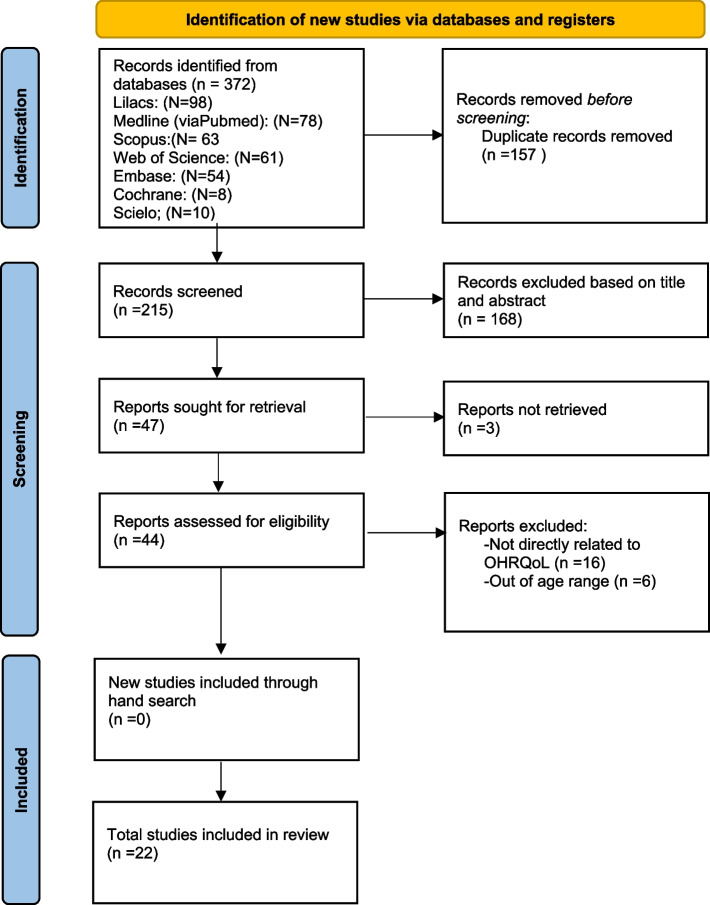
Flowchart of the search and article selection process, adapted from PRISMA guidelines

The search strategy found 362 articles. Only 22 systematic reviews were included, as shown in Fig. 1.

### Characteristics and methodological quality of eligible studies

This systematic umbrella review found 22 eligible articles. Only one article was written in Portuguese [26], and 21 studies were written in English, between 2009 and 2021. Malocclusion was the most collected variable in the studies [9, 19, 27–30] and traumatic dental injury (TDI) [31–35]. All systematic reviews have search criteria, eligibility criteria and study characterization. However, different quality assessment methods were used. The most cited method was the PRISMA [9, 27–29, 31–33, 35–44]. However, seven systematic reviews showed no risk of bias in the analyzed studies [9, 26, 30, 36, 43–45] and 10 studies did not perform meta-analysis [9, 26, 27, 30, 36, 38, 41, 43–45].

Oral conditions, characteristics of the selected studies and the OHRQoL measurement instruments can be found in the supplementary material of this article.

The methodological quality of the systematic reviews included (Table 1), based on the criteria proposed by AMSTAR 2, considered 10 articles of critically low quality [9, 27–30, 34, 36, 40, 45], 10 articles of low quality [29, 31, 33, 36–38, 41–44, 46], one systematic review of moderate quality [39], and one of moderate/high quality [34].

**Table 1:** Assessment of systematic reviews using AMSTAR 2 checklist

AMSTAR 2 question lists	Dimberg et al., 2014 [26]	Lopez et al., 2019 [27]	Zaror et al., 2018 [31]	Malele-Kolisa et al., 2019 [32]	Aimée Nicole et al., 2019 [36]	Barasuol et al., 2020 [37]	Lattanzi et al., 2019 [38]	Javidi et al., 2017 [39]
1.Did the research questions and inclusion criteria for the review include PICO components?	+	+	+	+	+	+	+	+
2. Did the report of the review contain an explicit statement that the review methods were established prior to the conduct of the review and did the report justify any significant deviations from the protocol?	+	?	-	?	?	?	?	-
3. Did the review authors explain their selection of the study designs for inclusion in the review?	+	+	+	+	+	+	+	+
4. Did the review authors use a comprehensive literature search strategy?	+	+	+	+	+	+	+	+
5. Did the review authors perform study selection in duplicate?	+	+	+	+	+	+	+	+
6. Did the review authors perform data extraction in duplicate?	+	+	+	+	+	+	+	+
7. Did the review authors provide a list of excluded studies and justify the exclusions?	+	+	+	+	+	+	+	+
8. Did the review authors describe the included studies in adequate details?	+	+	+	+	+	+	+	+
9. Did the review authors use a satisfactory technique for assessing the risk of bias (RoB) in individual studies that were included in the review?	-	+	+	+	+	+	+	+
10. Did the review authors report on the sources of funding for the studies included in the review?	-	-	-	-	-	-	-	-
11. If meta-analysis was performed did the review authors use appropriate methods for statistical combination of results?	?	-	-	+	+	+	?	+
12. If meta-analysis was performed, did the review authors assess the potential impact of RoB in individual studies on the results of the meta-analysis or other evidence synthesis?	?	-	-	+	+	+	?	+
13. Did the review authors account for RoB in individual studies when interpreting/ discussing the results of the review?	-	-	-	+	+	+	+	+
14. Did the review authors provide a satisfactory explanation for, and discussion of, any heterogeneity observed in the results of the review?	?	-	-	+	+	+	+	+
15. If they performed quantitative synthesis did the review authors carry out an adequate investigation of publication bias (small study bias) and discuss its likely impact on the results of the review?	+	-	-	-	-	-	?	-
16. Did the review authors report any potential sources of conflict of interest, including any funding they received for conducting the review	-	-	-	+	-	-	-	-
Review quality	Critically Low	Low quality	Critically Low	Low quality	Low quality	Low quality	Moderate	Critically Low
AMSTAR 2 question lists	Alrashed et al., 2020 [40]	Zhou1 et al., 2014 [28]	Silva Rodrigues et al., 2018 [45]	Mandava et al., 2021 [41]	Yactayo-Alburquerque et al., 2021 [9]	Milani et al., 2021 [46]	Bezerra et al., 2019 [33]	Kumar et al., 2014 [42]
1.Did the research questions and inclusion criteria for the review include PICO components?	+	+	+	+	-	+	+	-
2. Did the report of the review contain an explicit statement that the review methods were established prior to the conduct of the review and did the report justify any significant deviations from the protocol?	-	?	-	?	-	-	?	-
3. Did the review authors explain their selection of the study designs for inclusion in the review?	+	+	+	+	+	+	+	?
4. Did the review authors use a comprehensive literature search strategy?	+	+	+	+	+	+	+	+
5. Did the review authors perform study selection in duplicate?	+	+	+	+	+	+	+	+
6. Did the review authors perform data extraction in duplicate?	+	+	+	+	+	+	+	+
7. Did the review authors provide a list of excluded studies and justify the exclusions?	+	+	+	?	+	+	+	+
8. Did the review authors describe the included studies in adequate details?	+	+	+	+	+	+	+	+
9. Did the review authors use a satisfactory technique for assessing the risk of bias (RoB) in individual studies that were included in the review?	+	+	+	+	-	+	+	+
10. Did the review authors report on the sources of funding for the studies included in the review?	-	-	-	-	-	-	-	-
11. If meta-analysis was performed did the review authors use appropriate methods for statistical combination of results?	+	?	?	?	?	+	+	?
12. If meta-analysis was performed, did the review authors assess the potential impact of RoB in individual studies on the results of the meta-analysis or other evidence synthesis?	+	?	?	?	?	+	+	?
13. Did the review authors account for RoB in individual studies when interpreting/ discussing the results of the review?	+	-	+	-	-	+	+	+
14. Did the review authors provide a satisfactory explanation for, and discussion of, any heterogeneity observed in the results of the review?	+	-	+	-	-	+	+	+
15. If they performed quantitative synthesis did the review authors carry out an adequate investigation of publication bias (small study bias) and discuss its likely impact on the results of the review?	-	?	?	?	?	+	-	?
16. Did the review authors report any potential sources of conflict of interest, including any funding they received for conducting the review	-	-	-	-	-	-	-	-
Review quality	Critically Low	Critically Low	Low quality	Low quality	Critically Low	Low quality	Low quality	Low quality
AMSTAR 2 question lists	Kragt et al., 2015 [43]	Ferrando-Magraner et al., 2019 [29]	Liu et al., 2009 [44]	Oliveira et al., 2013 [25]	Antunes et al., 2020 [34]	Moghaddam et al., 2020 [30]		
1.Did the research questions and inclusion criteria for the review include PICO components?	-	-	-	+	+	+		
2. Did the report of the review contain an explicit statement that the review methods were established prior to the conduct of the review and did the report justify any significant deviations from the protocol?	-	-	-	?	-	+		
3. Did the review authors explain their selection of the study designs for inclusion in the review?	+	+	+	+	+	+		
4. Did the review authors use a comprehensive literature search strategy?	+	+	+	+	+	+		
5. Did the review authors perform study selection in duplicate?	+	+	+	+	+	+		
6. Did the review authors perform data extraction in duplicate?	+	+	+	+	+	+		
7. Did the review authors provide a list of excluded studies and justify the exclusions?	+	+	+	+	+	+		
8. Did the review authors describe the included studies in adequate details?	+	+	+	+	+	+		
9. Did the review authors use a satisfactory technique for assessing the risk of bias (RoB) in individual studies that were included in the review?	+	+	-	-	+	+		
10. Did the review authors report on the sources of funding for the studies included in the review?	-	-	-	-	-	+		
11. If meta-analysis was performed did the review authors use appropriate methods for statistical combination of results?	+	?	?	?	+	+		
12. If meta-analysis was performed, did the review authors assess the potential impact of RoB in individual studies on the results of the meta-analysis or other evidence synthesis?	+	?	?	?	+	+		
13. Did the review authors account for RoB in individual studies when interpreting/ discussing the results of the review?	+	+	-	-	+	?		
14. Did the review authors provide a satisfactory explanation for, and discussion of, any heterogeneity observed in the results of the review?	+	+	+	+	+	+		
15. If they performed quantitative synthesis did the review authors carry out an adequate investigation of publication bias (small study bias) and discuss its likely impact on the results of the review?	-	?	?	?	-	+		
16. Did the review authors report any potential sources of conflict of interest, including any funding they received for conducting the review	-	-	-	-	-	+		
Review quality	Critically Low	Low quality	Critically Low	Critically Low	Critically Low	Moderate/high		

Description

+ : Yes

-: No

?: Not mentioned

The Table 2, provides important characteristics of the selected studies.

**Table 2:** Characteristics of studies select

Referene	Article	Year Publication	Magazine	Country	Quality Analyses	Independent Variable	Nº of articles found in databases	Nº of articles incluides	Index Outcome	Maximum – Minimum variation Sample Size the studies	Maximum – Minimum variation Age Range	Conclusion	Metanálise
Kragt et al., 2015 [43]	The impact of malocclusions on oral health-related quality of life in children—a systematic review and meta-analysis	2015	Clin Oral Invest	Netherland	PRISMA	Malocclusion	3837	40	ECOHIS, CPQ 8–10, CPQ11-14, OHIP-14, COHIP, CS-OIDP, OASIS	121—5445	Mean 3.5 -14.9	There is association between malocclusion with OHRQOL. Age of children and their cultural environment influence OHRQOL	No
Liu et al., 2009 [44]	The Impact of Malocclusion/Orthodontic Treatment Need on the Quality of Life	2009	Angle orthod	China	Oxford centre for evidence-based medicine/	Orthodontic Treatment	135	23	WHOQOL-BREF, SF-36, psychologic scales, CPQ 11–14, OHIP 14, Oral Aesthetic Subjective Impact, Scale, PIDAQ, Child OIDP, OIDP, SOHSI, orthognathic quality of life questionnaire	89—1675	8–30 Adult	Association modest between malocclusion/orthodontic treatment need and QHRQoL	No
Oliveira et al., 2013 [25]	Reported Impact of Oral Alterations on the Quality of Life of Adolescents: A Systematic Review	2013	Pesq Bras Odontoped Clin Integ	Brazil	GRADE AXIS	Oral conditions	593	13	OIDP, COQ11-14, ISF16, OHIP	204—1745	10—19	Studies showed negative impact OHRQoL with influence malocclusion, dental caries and traumatic dental	Yes
Antunes et al., 2020 [34]	Does traumatic dental injury impact oral health-related to quality of life of children and adolescents? Systematic review and meta-analysis	2018	Int J Dent Hygiene	Brazil	Oxford centre for evidence-based medicine	Traumatic dental	689	11	ECOHIS and CPQ11-14	192—1632	10–14	Children under age 10 was significant in the symptom domain. Adolescent under age 11 to 14 was significant in the every domain	No
Lattanzi et al., 2019 [38]	Efects of oral health promotion programmes on adolescents’ oral health-related quality of life: a systematic review	2019	Int J Dent Hygiene	Brazil	PRISMA	oral health promotion programmes (OHPP)	2343	4	Child-OIDP	50 -1906	10—19	Studies showed positive effects of OHPP on adolescentes OHRQoL with low methodological quality	No
Liu et al., 2009 [44]	The Impact of Malocclusion/Orthodontic Treatment Need on the Quality of Life	2009	Angle orthod	-	Oxford centre for evidence-based medicine	Malocclusion\orthodontic	135	23	CPQ 11–14, COHIP, Child-OIDP, WHOQOL-BREF, SF-36, psychologic scale, orthognathic quality of life questionnaire OIDP, PIDAQ, OIDP, OHIP-14, Oral aesthetic subjective impact scale	-	8-adult	Association modest between malocclusionqorthodontic treatment need and OHRQoL	No
Magraner et al., 2019 [29]	Oral health-related quality of life of adolescents after orthodontic treatment. A systematic review	2019	J Clin Exp Dent	Spain	PRISMA	Orthodontic Treatment	817	10	OHIP-14 and CPQ 11–14	27—374	11–25	There is a positive association between end treatment and OHRQoL	Yes
Alrashed et al., 2020 [40]	The relationship between malocclusion and oral health-related quality of life among adolescents: a systematic literature review and meta-analysis	2020	Eur J Orthod	Saudi Arabia	GRADE/AXIS	Malocclusion	530	11	OHIP-14, CPQ 11–14, CPQ 8–10, COHIP SF-19, ISF 16	248—1206	11–18	Adolescent with severe maocclusiom have worst levels OHRQoL. Effects malocclusion influence by age, culture andenvironment in the OHRQoL	Yes
Kumar et al., 2014 [42]	A systematic review of the impact of parental socio-economic status and home environment characteristics on children’s oral health related quality of life	2014	Health Qual Life Outcomes	Australia	PRISMA	-Status socioeconômico parental -Ambiente familiar -Status socioeconômico -Renda -Ocupação dos pais -Nível de educação dos pais -Idade dos pais -Local de origem dos pais -Status matrimonial dos pais -Relacionamento Cuidador – criança -Estrutura familiar -Número de habitantes por moradia -Número de irmãos -Uso de álcool e droga -Nível de conhecimento parental sobre saúde oral -Ansiedade ao atendimento odontológico	5646	36	Child-OIDP, OIDP by interviewing children, OHIP-14, Modified OHIP-SP	95 -1412	2—21	Children from families with high income, parental educa- tion and family economy had better OHRQoL	Yes
Milani et al., 2021 [46]	Impact of traumatic dental injury treatment on the Oral Health-Related Quality of Life of children, adolescents, and their family: Systematic review and meta-analysis	2020	Dent Traumatol	-	GRADE	Traumatic dental	414	6	ECOHIS, FIS 8-10 s, cpq8-10, CPQ 11–14, SOHO, P-CPQ 8–10, CPQ 11–14	-	2–20	Treatment of traumatic dental injuries reduces the impact on the OHRQoL with low evidence	Yes
Bezerra et al., 2019 2019 [42]	Does bruxism impact the quality of life of children and adolescents? A systematic review and meta-analysis	2019	J Public Health	Brazil	GRADE	Bruxism	130	3	Oidp, coq11-14, ISF16, Ohip, AUQUEI, PedsQL4.0, ECOHIS, CSHQ	21—462	3–14	Bruxism does not impact quality of life in children and adolescentes	Yes
Zhou et al., 2014 [28]	The impact of orthodontic treatment on the quality of life a systematic review	2014	Bmc Oral Health	China	Oxford centre for evidence-based medicine	Orthodontic Treatment	204	11	OHQoL-UK, cs-OIDP, OHIP14, CPQ11-14	118- 1675	7–33	There is association modest between orthodontic treatment and quality of life	No
Mandava et al., 2021 [41]	Impact of self-esteem on the relationship between orthodontic treatment and the oral health-related quality of life in patients after orthodontic treatment – a systematic review	2021	Med. Pharm. Rep	Índia	Cochrane risk of bias tool/ Newcastle ottawa scale modificada	Orthodontic Treatment	7688	28	OIDP and OHIP-14, PIDAQ, CPQ8-10, 11–14,	27–1675	11–25	OHRQoL and SE in children. OHRQoL also increased in adolescents and adults. However, there is a weak correlation between SE and OHRQoL. More evidence-based studies are needed to analyze the relationship	
Javidi et al., 2017 [39]	Does orthodontic treatment before the age of 18 years improve oral health-related quality of life? a systematic review and meta-analysis	2017	Ajo-Do	United Kingdon	Cochrane collaboration's	Orthodontic Treatment	3359	6	CPQ11-14, OHIP14, OIDP	27–374	11–30	Orthodontic treatment during child- hood or adolescence leads to moderate improvements in the emotional and social well-being dimensions of OHRQoL, although the evidence is of low and moderate quality	
Rodrigues et al., 2018 [45]	Does dental agenesis have an impact on ohrqol of children, adolescents and young adults? a systematic review	2018	Acta Odontol. Scand	Brazil	PRISMA	Agenesia Dental	178	3	CPQ 11–14, OIDP, P CPQ,CS OIDP	116–163	11–17	No articles were found that had evaluated children and adolescent. Only one was found to have a greater impact in the adolescent agenesis group with statistical diferences	No
Yactayo-Alburquerque et al., 2021 [9]	Impact of oral diseases on oral health-related quality of life: a systematic review of studies conducted in latin america and the caribbean	2021	Plos One	France	PRISMA	Oral conditioms Malocclusion Dental traumatic Periodontal disease Temporo mandibular dysfunction, salivar gland pathology, cleft lip and palate, tooth decay	3310	40	CPQ 11–14, ECOHIS and B-ECOHIS	100–1614	1–64	Studies in LAC report a negative impact of diseases on OHRQoL: tooth decay, malocclusion, xerostomy. muscle disorder, severe periodontal disease	No
Moghaddam et al., 2020 [30]	The Association of Oral Health Status, demographic characteristics na socioeconomic determinants with Oral health-related quality of life among children: a systematic review and Meta-analysis	2020	BMC Pediatrics	Iran	PRISMA—The Joanna Briggs Institute (JBI)	Oral health promotion strategies	4254	11	-	103–1134	3–12	Oral health promotion strategies to improve children’s OHRQoL should consider the social and environmental where they live as well their oral health status	
Dimberg et al., 2014 [26]	The impact of malocclusion on the quality of life among children and adolescents: a systematic review of quantitative studies	2014	Eur J Orthod	Maloclusão	GRADE	Malocclusion	1156	6	CPQ, OHIP, OIDP	1204–225	8–15	There is strong association between malocclusions and OHRQOL, predominantly in the dimensions of emotional and social wellbeing	No
Lopez et al., 2019 [27]	Impact of uncomplicated traumatic dental injuries on the quality of life of children and adolescents: a systematic review and meta-analysis	2019	BMC Oral Health		PRISMA	Traumatic Dental	712	26	SOHO, ECOHIS, OIDP, CPQ11-14, CPQ8-10, OHIP	192- 7328	1–14	Uncomplicated TDIs do not have a negative impact on the OHRQoL of children and adolescents	Yes
Zaror et al., 2017 [31]	Impact of traumatic dental injuries on quality of life in preschoolers and schoolchildren: a systematic review and meta-analysis	2017	Community Dent. Oral Epidemiol	Spain and Chile	PRISMA	Traumatic Dental	237	26	SOHO, ECOHIS, CHILD OIDP, CPQ11-14, CPQ8-10, OHIP E OIDP, ISF10, ISF16	1–15	50–1528	Traumatic dental injuries have a negative impact on OHRQoL of both preschoolers and schoolchildren	Yes
Aimée et al., 2019 [36]	Responsiveness of oral health-related quality of life questionnaires to dental caries interventions: systematic review and meta-analysis	2018	Caries res	Brazil	GRADE	Dental Caries	3643	14	COHQoL P-CPQ and FIS, e OHIS, SOHO, CHILD OIDP, CPQ 8–10 e CPQ 11–14	32–335	3–50,8	There is low evidence that the OHRQoL of children and adolescents improved following caries inter- vention procedures	No
Barasuol et al., 2020 [37]	Association between dental pain and oral health-related quality of life in children and adolescents: asystematic review and meta-analysis	2019	Community Dent. Oral Epidemiol	Brazil	The fowkes and fulton checklist	Dental pain	3474	14	SOHO-5, B-ECOHIS and Child- OIDP	132–1052	children and adolescents up to 19 years of age	There is low evidence that dental pain has a negative impact on OHRQoL	Yes
Malele-Kolisa et al., 2019 [32]	Systematic review of factors influencing oral health-related quality of life in children in africa	2019	Afr. J. Prim. Health Care Fam. Med	South Africa	Checklist for cross-sectional, random controlled trials and cohort studies, by the joanna briggs institute	Oral condition, dental caries, socioeconomic status, contextual social determinant	337	15	ECOHIS, OIDP, CPQ 8–10 E 11–14	92–2678	6 month-21 years old	There is association between individual factors such as children’s psyche and oral problems. Dental caries was not impacto in OHRQoL	No

### Oral conditions and OHRQoL in adolescents

#### Impact of malocclusion on OHRQoL

The studies who avaliate occlusal disorders, concluded that this pathology have a negative impact on OHRQoL in adolescents [9, 19, 27–30]. There are divergences related to the degree of severity of impact on OHQoL. The emotional and social domains obtained higher scores when compared to the functional domains. Aesthetics and satisfaction with appearance have the greatest impact on OHQoL [34]. The studies evaluated incisal crowding, maxillary anterior irregularity ≥ 2 mm, and overjet ≥ 5 mm.

Two systematic reviews note that only cross-sectional studies were included, which cannot record causality [28, 29]. In addition, it was inferred that adolescents with malocclusion have a greater impact on OHRQoL when compared to children. Adolescents who had never received orthodontic treatment had a greater impact on quality of life compared to patients who had already completed treatment [34, 40, 44–46]. Lastly, the study ndicate that the degree of negative impact on OHRQoL is directly proportional to the need for orthodontic treatment and its consequent aesthetic impairment [34].

### TDI and OHRQoL

The impact of dental trauma sequelae on OHRQoL in adolescents was observed in five systematic reviews [31–35]. The studies [31–35] show that uncomplicated traumatic injuries do not have a negative impact on the OHRQoL of adolescents. The negative effect is greater when it involves pulp exposure or darkening of the dental element [32], and the age group from 11 to 14 years is the most affected [35]. Adolescents report difficulty smiling, eating, socializing, presence of pain, difficulty in chewing [34].

Treatment of TDI reduces the negative impact on OHRQoL in adolescents, based on parental perception [33]. Individuals with a fractured tooth, who do not receive treatment, have a four times greater risk of reporting an impact on OHRQoL when compared to the group without trauma [34]. Negative self-perception remains after tooth restoration.

### Dental caries, periodontal disease, toothache, dental erosion, agenesis, edentulism, bruxism, DTM and OHRQoL

The impact of dental caries on OHRQoL was addressed in five systematic reviews [9, 26, 34, 36, 37]. Three articles report that the greater the severity of the carious lesion, the worse the impact on OHRQoL in adolescents [9, 26, 34]. As well as individuals with severe periodontitis had worse OHRQoL scores [9]. The association between caries and periodontal disease was demonstrated in an sistematic review. And the operative treatment of caries lesions has a positive effect on OHRQoL, despite the low quality of evidence [9]. Toothache, DMT and tooth loss have a high impact on OHRQoL in adolescentes [26]. While dental erosion and bruxism have not been shown to impact the quality of life of adolescents [26]. In contrast, tooth agenesis does not have enough scientific evidence to support a relationship between OHRQoL [41].

### Impact of health determinants on OHRQoL

Only three systematic reviews assessed the impact of oral health determinants on OHRQoL [36, 39, 43]. It was observed that health promotion programs have a positive effect on OHRQoL. The reduction of oral problems and increased satisfaction with oral health in the development of daily activities such as chewing, brushing, talking, smiling and sleeping are reported in studies [39].

Another finding demonstrated that having parents who can provide dental care and safe housing are positive predictors for OHRQoL. The systematic review reports factors that physical disability, visual impairment, mental disorders, poor diet and irregular brushing negatively impact OHRQoL. While the influence of religion and age on OHRQoL is unknown [36].

Socioeconomic factors related to the area of residence, satisfaction with oral health and dental care were shown to be directly proportional to the OHRQoL outcome [36]. The parental socioeconomic factor and family environment also influenced OHRQoL [43]. Adolescents from families with higher incomes and higher levels of maternal education have better OHRQoL scores [34]. Being an only child, growing up in your nuclear family or family structure, household conditions, and number of people per household and maternal age are predictors of better OHRQoL [43]. While parental occupation, marital status, and the family provider being the mother or direct caregiver were not factors capable of impacting OHRQoL [43]. It is noteworthy that the parent’s place of origin, place of study, deleterious habits in the family, resistance to dental care on the part of the mother and use of dental care services do not have strong evidence.

### Assessment instruments and OHRQoL

Systematic reviews [26–33, 36–39, 41–43, 45, 46] report the use of different OHRQoL assessment instruments in adolescents. We found 21 questionnaires used in different study methodologies. Child Perceptions Questionnaire (CPQ), Oral Impact on Daily Performances (OIDIP), Early Childhood Oral Health Impact Scale (ECOHIS) and The Oral Health Impact Profile (OHIP), Child Oral Impacts on Daily Performances (Child OIDP) were the most frequent measurement systems. The supplementary material to this article contains the different OHRQoL measurement instruments used in the 22 studies [9, 26–34, 36–46] 46 review.

## Discussion

Many systematic scientific reviews on this topic are found in the literatue, in the process of writing this article. However, it is essential to consider the methodological rigor of the studies in order to expand scientific knowledge. Thus, supporting decision-making and generating data for the implementation of health strategies and programs focused on specific and vulnerable populations. This is the first systematic umbrella review to provide an overview of factors that impact HRQoL in adolescentes between 10 and 19 years of age. The main findings show that dental caries, malocclusion [9, 26–30], TMD [9], dental trauma (TDI) [32, 34, 35], poor brushing [36], toothache [26], periodontal disease [34] and edentulism [9] negatively affect the quality of life of adolescents. The need for orthodontic treatment [26, 34] and the completion of orthodontic treatment [40, 44–46] also influence behavior and self-perception related to oral health in adolescents. This can be explained by the changes in adolescence and the increase in aesthetic perception, involving social, behavioral and psychological factors [5]. In addition, we can see that pain and aesthetics cause greater demand for dental care, causing financial expenses and impact on quality of life and parents tend to report the worst impact.

Another important finding is related to social determinants, such as demographic and socioeconomic factors. Being an only child, growing up in your nuclear family, housing area and security, level of maternal education, access to dental care and the performance of health promotion programs are directly proportional to OHRQoL in adolescents [36, 39, 43, 47]. This can be explained by the level of information and awareness, which favors similar behaviors. People with higher educational level tend to make better health choices.

As previous studies reported, low socioeconomic status, poor social support, negative oral health beliefs and lower levels of protective psychosocial factors were significantly associated with unhealthy behaviours and poor HRQoL in adolescentes [4]. Moreover, this indicators can be used to identify the risk of impaired OHRQoL already at the beginning of adolescence [48]. This systematic review observed that the place of origin of those responsible and use of services does not have sufficient scientific evidence. Parental occupation, marital status and the family provider being the mother or direct caregiver were not able to impact adolescents' self-perception on OHRQoL [43]. Perhaps, these findings can be explained by the adolescents' self-perception related to group acceptance and social support from the environment they live in. There are two theories that can explain the process: the psychosocial conceptual model and the lifetime risk accumulation model. Family or social groups tend to present the same health behaviors and this can have a negative or positive influence throughout life. The socio-environmental context and health choices throughout life can influence the development of diseases, including the socioeconomic level of the adult individual.

It should also be said that the subject's condition of life is determined by the position he occupies in space in relation to the type of power or capital obtained. Thus, economic capital (income), while it can generate specific risks such as occupational ones, symbolizes greater access to care and living conditions, allowing better coping with the illness process; cultural capital (level of education) allows access to knowledge about the risks of becoming ill and prevention; symbolic capital (prestige, personal/professional recognition) is related to the subjective dimension of people's satisfaction with life, making them more normative in their environment; and social capital (social cohesion) concerns a set of elements of social organization, such as mutual trust, solidarity and civic engagement, which facilitate the coordination and cooperation of collective actions to achieve mutual benefits. Therefore, it can be said that exposure to different risks depends on how the individual places himself in different fields, as well as the relationships resulting from this position.

Biological data such as skin color and age have no concrete evidence about this impact in the OHRQoL. These factors could establish a strong correlation between demographic and socioeconomic factors. In the literature, this relationship is conflicting [48, 49]. There is great heterogeneity in age-related data collection. There is no standardized assessment. The cognitive understanding of a 5-year-old child is different from that of a 12-year-old, so it would be impossible to apply the same instrument to both age groups simultaneously. Thus, the questions are adapted and validated for the age group according to the cognitive needs of each one. It should be noted that self-perception and children's cognitive health are considered age-dependent and the result of continuous cognitive, emotional, social and language development. Therefore, it is important to obtain information from the child's parents or guardians in order to obtain complete information, thus obtaining an effective questionnaire, that is, capable of measuring the impact of oral conditions on the quality of life related to the oral health of children.

Another important aspect is the method of evaluating the OHRQoL outcome. Different measurement instruments were found in the 22 systematic reviews included in this study, which makes data analysis and results interpretation difficult. In this context, a large number of instruments that assess the impact of oral conditions on oral health-related quality of life have been produced and validated worldwide, with the aim of providing greater accuracy to individual and collective assessments. These instruments have become fundamental to complement clinical measures, but there is little guidance for the proper selection of these instruments, since there are principles to be followed.

In addition, the selected articles investigated different independente variable. Same variable presented different methods of observation and evaluation. Statistical approaches were also different, including univariate and multivariate regression. Therefore, the heterogeneity in the process of producing evidence and in the methodology proposed by the studies is highlighted. Likewise, differences were observed in the sampling according to age and gender. Age, developmental level and gender influence and affect the well-being of young people [34, 50]. Even the sample size of the different studies included in the systematic reviews demonstrate methodological flaws and limitations, which can lead the reader to misinterpret the results.

It was observed that only one article was considered of moderate/high quality [34] and one article of moderate Quality [39], ten had critically low Quality [9, 27–30, 32, 35, 38–40, 45], and ten others had low quality [29, 31, 33, 36–38, 41–44, 46], according to the AMSTAR 2-based method. This made it difficult to carry out the meta-analysis of this systematic review.

AMSTAR 2 was the instrument used in the present study, with the objective of critically qualifying the reviews. The tool has a robust and adequate method for evaluating sistematic reviews [21, 23, 24] and was deprecated from the RoB 2 (Cochrane risk-of-bias tool), as it focuses its analysis criteria on the field of randomized trials [24]. In addition, a trend towards the adoption of AMSTAR 2 as a methodological quality assessment tool was observed in the umbrella reviews published in the medical and dental field [35, 51–54].

Three initially selected systematic reviews were later excluded due to lack of information, as the corresponding author did not respond to our contact to provide the necessary information. This fact can also be pointed out as a limiting factor, since the purpose of the umbrella review is to analyze the totality of sistematic reviews relevant to a given topic. It is impossible to ignore the fact that these studies could add new evidence, corroborate or refute the results obtained.

Umbrella systematic reviews are a recent modality of study, with no established conduction protocols. It is prudent to infer that this is a compilation of the above information organized in a concise manner. The search strategy was judicious, however there is a scarcity of studies with high quality on quality of life related to oral health. Important aspects such as tooth loss, agenesis, bruxism, TMD are neglected and little studied, despite the complaints of patients in the clinical routine.

The inclusion of gray literature was considered and a simple search strategy was even run in Opengrey and Google Scholar databases. As a result, an exorbitant number of works was obtained that did not meet the inclusion criteria, such as: theses, reports, annals and critical reviews. So, it was decided not to include the gray literature, despite not knowing the real harm in obtaining new evidence.

## Conclusion

This systematic umbrella review found that dental caries, malocclusion, temporomandibular disorders, dental trauma, poor brushing, toothache, periodontal disease, and edentulism vahe a negative impact on oral health-related quality of life. In addition, social determinants, such as demographic and socioeconomic factors, like being an only child, growing up in your nuclear family, housing area and security, level of parental education, access to dental care and the performance of health promotion programs are directly proportional to OHRQoL and self-perception in adolescentes. Important aspects like gender and skin color did not have their level of impact clarified. These findings are important to clarify what context can cause negative impact in adolescents daily lives. Armed with this knowledge, strategies and public policies focused on this age group, will be assertive.

## Data Availability

Any data that support the findings of this study are available from the corresponding author, upon reasonable request.
